# Tracking Chinese Online Activity and Interest in Osteoporosis Using the Baidu Index

**DOI:** 10.7759/cureus.57644

**Published:** 2024-04-05

**Authors:** Jianjun Wu, Yugeng Zheng, Xianchan Lin, Shi Lin, Hongxing Huang

**Affiliations:** 1 Third School of Clinical Medicine, Guangzhou University of Chinese Medicine, Guangzhou, CHN; 2 College of Pharmacy, Shenzhen Institutes of Advanced Technology of the Chinese Academy of Science, Shenzhen, CHN; 3 Department of Pediatric Orthopedics, Foshan Hospital of Traditional Chinese Medicine, Foshan, CHN; 4 Department of Clinical Medicine, Guangdong Maoming Health Vocational College, Maoming, CHN; 5 School of Nursing, Guangzhou University of Chinese Medicine, Guangzhou, CHN; 6 Osteoporosis Research Institute, Third Affiliated Hospital of Guangzhou University of Chinese Medicine, Guangzhou, CHN

**Keywords:** search behavior, baidu, internet, osteoporosis, baidu index

## Abstract

Introduction

China's most widely used online search engine, Baidu (Baidu, Inc., Beijing, China), has developed a data collection and analysis tool called the Baidu Index for tracking Internet search trends. The purpose of this study was to examine the utility of the Baidu Index in tracking online osteoporosis information-seeking behavior and comprehending the traits and concerns of the Chinese population.

Methods

We used the search term "osteoporosis" for the Baidu Index for the years 2018-2022. The geographic and demographic distributions, search volumes, and demand maps were recorded.

Results

The popularity of the search term "osteoporosis" has increased over time. The search was mostly conducted among women aged 20-39 in northern China. The demand map revealed that the most significant concerns are related to the diagnosis, treatment, and etiology of osteoporosis.

Conclusion

The Baidu Index is a valuable tool for tracking online health information-seeking behavior among Chinese netizens. Online search trend data appears to reflect the geographic and demographic aspects of osteoporosis to a certain extent.

## Introduction

Osteoporosis is a skeletal disease in which bone strength decreases and fracture risk increases [1]. The United Nations (UN) General Assembly declared 2021-2030 the Decade of Healthy Aging, which underscores the importance of policymakers around the world focusing their policies on improving the lives of older people today and in the future [2]. The situation of osteoporosis in middle-aged and elderly people is grim due to the acceleration of aging, and the number of elderly people suffering from osteoporosis is increasing [3]. With the acceleration of urbanization and population aging in China and the widespread prevalence of unhealthy lifestyles, the prevention and control of osteoporosis in China is becoming increasingly grim [4,5].

With the rapid growth of the Internet, mass information has gradually penetrated human life. According to the 50th China Statistical Report on Internet Development, the number of search engine users in China reached 821 million as of June 2022, or 78.2% of Chinese netizens [6,7]. At present, the dominant search engine in mainland China is Baidu (Baidu, Inc., Beijing, China). Nearly 92.1% of Internet users acknowledge Baidu as their preferred and dominant search engine [8,9]. By examining these online search trend data, it is possible to represent the behavior and interest patterns of Internet users looking for health-related information at the population level.

As a tool for gathering and analyzing Internet search trends, the Baidu Index offers an up-to-date information source to evaluate public discussion, epidemiology, and the characteristics of different diseases [10]. To the best of our knowledge, there has been no online Chinese research on osteoporosis. We conducted this study to further comprehend the characteristics and priorities of osteoporosis patients, as well as to determine whether interest search trend data accurately reflect the burden of osteoporosis. This study intends to investigate the relevance of online search data in studying Internet users' behavior while seeking information on osteoporosis.

This article was previously posted to the medRxiv preprint server on November 3, 2023 [11].

## Materials and methods

Data collection

The study was conducted at the Guangzhou University of Chinese Medicine, Guangzhou, China. Data were sourced from the Baidu Index (https://index.baidu.com/), a comprehensive big data-sharing service leveraging user behavior data from Baidu search queries (Appendix 1). This platform facilitates the analysis of keyword search trends, user demand shifts, media opinion trends, and digital consumer characteristics, offering insights from both consumer and industry perspectives.

Data types and retrieval processes

Search Index

This index quantifies user searches on the Baidu platform, using specific keywords as statistical entities. It includes detailed scientific analyses and weighted sums of search frequencies for each keyword, further categorized by the search origin: personal computers (PC) or mobile devices. The data retrieval process was standardized to ensure consistency across time frames and devices, with specific filters applied to isolate relevant searches.

Media Index

Derived from Baidu's intelligent distribution algorithms and recommended content, this index aggregates user interactions such as reading, commenting, forwarding, tagging, and disliking. It reflects the engagement and dissemination of content related to the specified keywords.

Geographical Distribution

This aspect of the data illustrates the distribution of search activity by province and municipality, offering insights into regional interests and preferences regarding the keywords.

Population Attributes

Data on the age and gender distribution of users searching for the specified keywords were collected to analyze demographic patterns in information-seeking behavior.

Keyword and data collection specifics

For this study, "osteoporosis" was the keyword of interest. Data on search and media indices was collected from 2018 to 2022, encompassing five years. Due to the limitations of the Baidu Index tools and analytical requirements, data were recorded weekly and then aggregated quarterly and annually for comprehensive analysis. This approach addressed the need for detailed temporal insights while accounting for the granularity of the available data.

## Results

**Figure 1:**
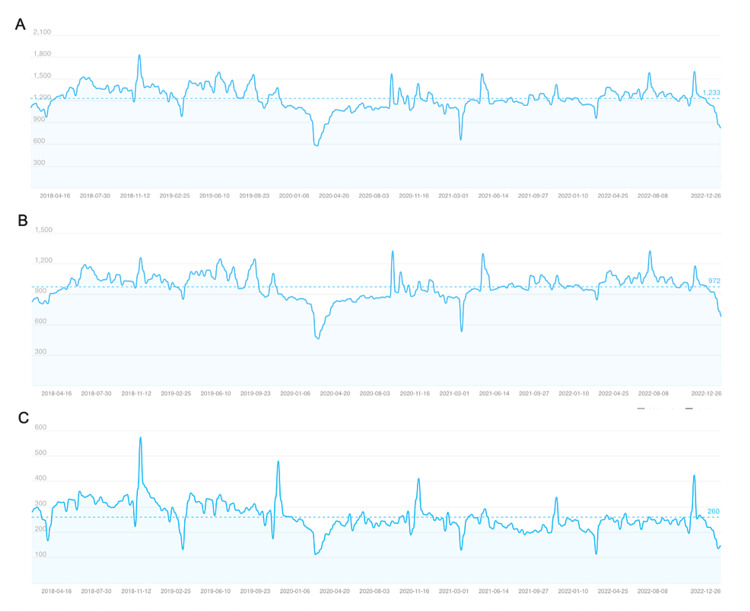
The "osteoporosis" search volume index on Baidu in China from 2018 to 2022. This figure displays the volume of searches on average, including the index of overall search trends, the mobile phone search index, and the personal computer search index. A: Overall search trends show a relatively stable volume with minor fluctuations over time; B: The mobile phone search index indicates slight variability but general stability in search volume; C: The personal computer search index showcases a more pronounced variability and a significant drop in search volume.

**Figure 2:**
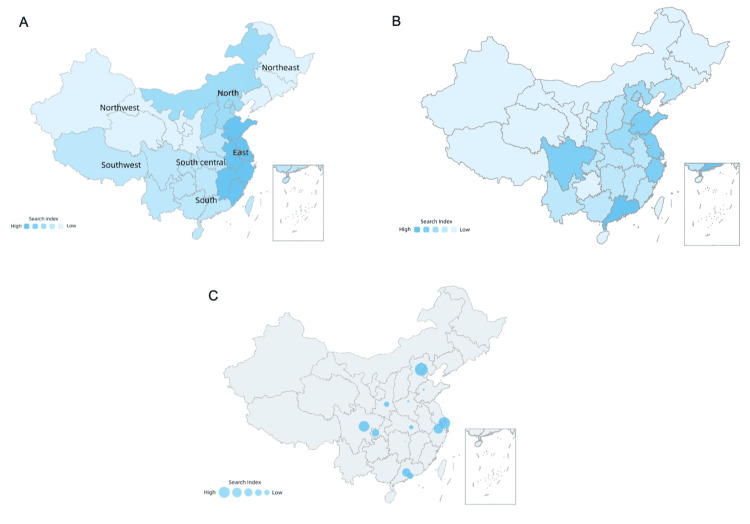
Maps from the 2018 to 2022 Baidu Index for "osteoporosis" by area, province, and city. A: Regional distribution highlights search volumes across China's main geographic areas, showing varying levels of interest; B: Provincial breakdown reveals specific areas with higher search activities; C: City-level analysis indicates concentrated search interest in certain urban areas, represented by circle sizes. This figure is based on data from the Baidu Index (http://index.baidu.com). It is part of a preprint made available under a CC-BY-NC-ND 4.0 International license, with the copyright held by the authors, who have granted medRxiv a license to display the preprint in perpetuity [11].

**Figure 3:**
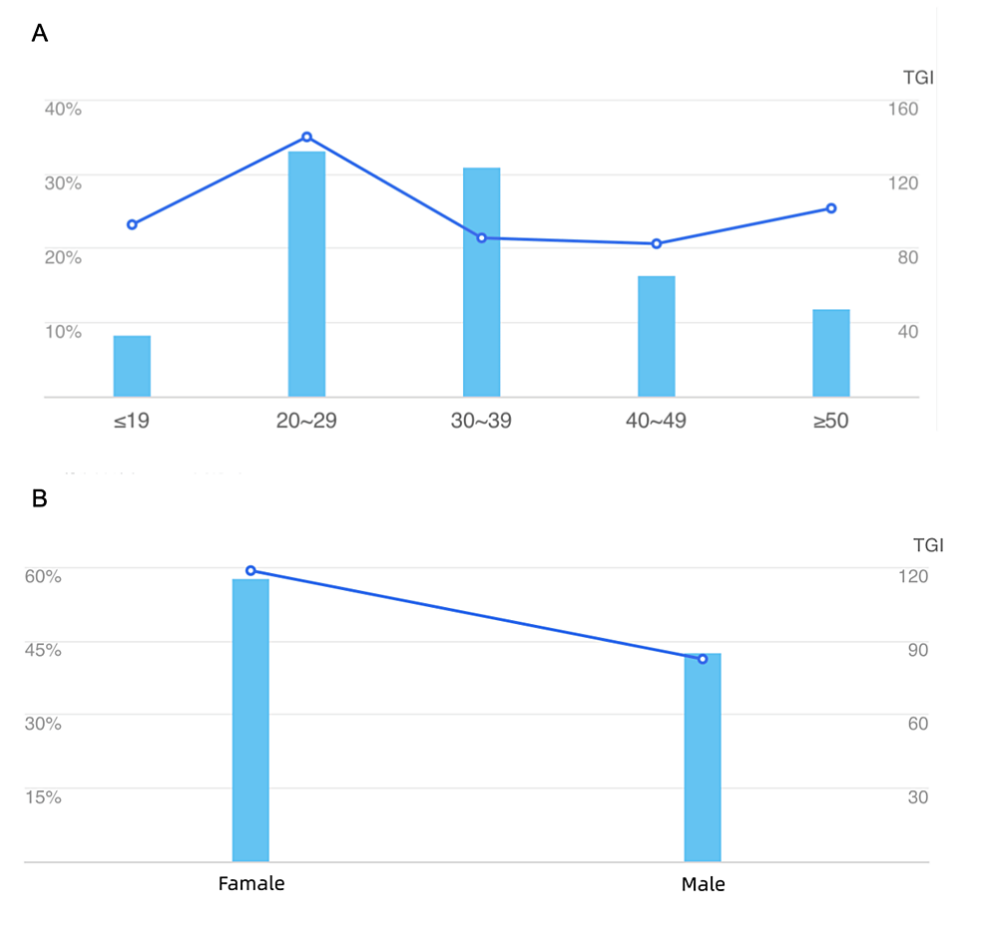
Demographic profiles by age and sex. A: Age-related search trends show the highest interest in the 20-29 age group, with a noticeable decline in older age groups. The target group index (TGI) demonstrates the relative search intensity across different age groups; B: Gender comparison in search trends highlights a significantly higher search interest among females compared to males, as well as the TGI indicates the proportional interest level.

Over the past five years, the interest in "osteoporosis" on Baidu has steadily increased, with distinct trends observed between mobile and personal computer searches. Mobile searches align with the overall Baidu Index's growth, while PC searches have declined annually (Figure 1). Geographic analysis of searches indicates higher volumes in socioeconomically developed areas (Figure 2). Demographically, the majority of queries come from individuals aged 20-39 and females (Figure 3).

Analysis of Baidu's search data reveals the top osteoporosis-related queries, emphasizing diagnosis, treatment options, dietary control, etiology, and associated diseases, with "What are the symptoms of osteoporosis?" leading the list (Table 1).

**Table 1:** Top 10 osteoporosis-related search terms by categories in the Baidu Index

Rank	Category	Search term
1	Diagnosis	“What are the symptoms of osteoporosis?”
2	Treatment options	“Calcium supplement”
3		“Calcitonin”
4	Diagnosis	“Bone density”
5	Etiology	“Calcium deficiency”
6	Treatment options	“Zoledronic acid”
7	Diet control	“What to eat for osteoporosis?”
8	Diagnosis	“Bone mineral density test”
9	Concomitant diseases	“Rib fracture”
10		“Vertebral compression fracture”

The fastest-growing osteoporosis-related search terms focus on treatment methods, dietary recommendations, and understanding the disease, with "How to treat osteoporosis" being the most searched term (Table 2). 

**Table 2:** Top 10 osteoporosis-related search terms that are growing quickly in the Baidu Index

Rank	Search term
1	How to treat osteoporosis?
2	Healthy bones
3	Symptoms of osteoporosis
4	What to eat for osteoporosis?
5	Bone mineral density
6	Treatment of femoral head necrosis
7	Causes of osteoporosis
8	Calcium loss
9	What foods contain high calcium?
10	Alendronate sodium

## Discussion

Over the past few decades, rapid economic growth has created space for policies to improve health and well-being; infectious diseases have gradually decreased; and chronic diseases have been effectively prevented. The average lifespan of people has increased considerably [12]. People worldwide are living longer. Every country in the world is experiencing growth in both the size and proportion of older people in its population. China is home to one-fifth of the world's older people, and little awareness exists concerning osteoporosis, which makes it challenging to determine the exact prevalence of osteoporosis and understand the characteristics, requirements, and demands of people with osteoporosis [13,14]. Fortunately, the combination of the Internet's extensive use and the public's growing dependence on search engines as sources of health-related information has produced tremendous results, especially during COVID-19 [15,16]. Search engine data may also complement and extend the current clinical and epidemiologic data [17,18]. User search behavior can become a new development direction in healthcare [19].

This study demonstrates the prospect of utilizing data on search engine trends to simulate the actual situation encountered by Chinese people with osteoporosis. In 2010, Google China Search Service was pulled out of mainland China, and the Baidu Index tracked public health and predicted diseases in China. The Chinese Center for Disease Control and Prevention (China CDC) and Baidu have developed a real-time disease prediction product using the Baidu Index, which can provide real-time influenza data as well as data on AIDS, hypertension, diabetes, and other diseases. In addition, as a potent addition to conventional detection methods, it is employed to forecast the epidemic trends of diseases [20]. Despite its promise, there has not been much use of the Baidu Index in medical research, and a large amount of data is still left to be gathered, especially for patients with skeletal diseases. There has never been an osteoporosis-related research project in China that uses the Baidu Index. Our research is the initial effort to examine Chinese online users' behaviors and interests around osteoporosis and confirmed that the hundred index data provided a measure of the epidemiology of osteoporosis diseases and public interest in treatment modalities.

The findings of this study demonstrated an increase in public awareness of osteoporosis over five years. This may be due to the increasing prevalence of osteoporosis, public health awareness, or Internet popularity, particularly for mobile phones. In particular, an increase in searches during the summertime of each year, when there is more sunshine and a higher average temperature, may be attributable to increased awareness of bone health [21]. Overwhelming evidence shows that sun exposure is beneficial in the prevention of osteoporosis [22]. Similarly, exposure to high temperatures promotes vitamin D3 synthesis in the human skin [23]. Furthermore, we discovered that while the personal computer search index for osteoporosis clearly exhibited a downward trend, the mobile search index did not, probably as a result of the development and broad use of the Internet and mobile device technology in the recent past. Considering the growing number of mobile devices being used by Internet users to seek health-related data about osteoporosis, health educators working to encourage people to adopt healthy habits and behaviors can more effectively direct target audiences with the help of social media for mobile devices.

Our analysis of regional variances revealed that, to a certain extent, the interest search trend of the Baidu Index may indicate the actual nationwide healthcare trend of osteoporosis. The amount of osteoporosis retrieved in northern China was much higher than that in southern China, which may reflect the geographical distribution pattern of osteoporosis incidence and prevalence in China, reflecting the influence of environmental differences on bone formation [24].

To investigate regional disparities, China was divided into seven conventional geographical regions. The seven regions were then ordered from large to small according to the magnitude of the search query, as shown in Figure 2 (east, north, southwest, south, south-central, northeast, and northwest). The reported prevalence of osteoporosis in urban areas is significantly higher than that in rural areas, according to the latest osteoporosis epidemiological study data by the China CDC [25]. Contrary to the study, significant variations between the stated osteoporosis prevalence at the urban and rural levels were observed in the Baidu Index search volume. In rural areas, awareness levels are lower than those in urban areas, which explains this. The top 10 provinces and cities with the highest search activity had floaty populations and higher socioeconomic levels. While this may suggest that the Baidu Index lacks the details necessary to assess epidemiologic osteoporosis trends at these scales, it is also possible that the apparent differences are the result of more economically developed regions with larger populations, a more complete Internet infrastructure, better health outcomes, and a higher level of public awareness. The Baidu Index offers standardized search statistics for search results provided by all users of Baidu, including both native and non-native inhabitants, and a cross-sectional assessment of the prevalence of osteoporosis in China centered on inhabitants. As a result, it is possible that reference prevalence may not accurately reflect the true prevalence of osteoporosis, as indicated by the Baidu Index. In addition, online health information gathering can allow increased healthcare engagement [26]. The 'prevalence' conveyed by Internet searches may be more significant in this regard.

Most search terms for "osteoporosis" came from females (57.57%) and the 20-39 age group (63.83%). Unlike osteoporosis, which is associated with postmenopausal and old age, the unusually large search volume for this age-related "osteoporosis" could be because such a group has easier access to the Internet. Although an increase in the prevalence of osteoporosis is positively correlated with age, a considerable number of low bone mass state and early stages of osteoporosis usually have no obvious clinical features [27]. Due to the lack of public awareness of the importance of osteoporosis prevention and the lack of prevention and treatment capacity for osteoporosis in primary healthcare institutions, the bone mineral density detection rate of Chinese residents is relatively low, and most residents do not take timely prevention and control measures at the initial stage of bone mass decline [25]. Another explanation may be that the 20-39 age group has more convenient access to the Internet, and as a group of children, parental discomfort occurs, resulting in a greater demand for osteoporosis-related information [28,29]. In addition, a higher target group index (TGI) indicates that female Internet users are more interested in osteoporosis-relevant information, which is consistent with the prevalence trend of osteoporosis and is equivalent to a higher volume of searches.

Healthcare professionals can use data from the Baidu Index to inform their teaching and clinical practice regarding the top 10 search phrases and the top 10 search terms with the highest rate of growth. Beyond conventional quantitative and qualitative metrics of disease surveillance, search term variations over time can provide insight into the attention and prioritization of patients in real-time. The findings revealed that the top three studies focused on the diagnosis, treatment, and etiology of osteoporosis. This shows that queries regarding diagnosis, cause, and treatment are prominent. These findings will help health professionals better understand the needs of the general population while conducting osteoporosis prevention initiatives.

The usefulness of the Baidu Index also has several constraints. This study was restricted to the Internet search engine Baidu, which solely uses terminology in Chinese and is located in China. Consequently, the data produced by other search engines was not evaluated in our study. Moreover, it is not possible to collect demographic information on Baidu users other than their age and gender (e.g., race, educational background, socioeconomic level, or other aspects connected to their use of Baidu). The Baidu Index might be biased by sampling because the personal data of Internet users is significantly biased towards groups with higher socioeconomic status and higher education levels. Additionally, certain significant demographic subgroups can be missed, and their search terms might not contain "osteoporosis.” For instance, "bone loss"-related search terms were not used in our investigation. As a result, our study may have underestimated the target osteoporosis population. The incentive for implementers to perform online searches is also unknown because the number of searches for osteoporosis may be influenced by patients, researchers, and healthcare professionals. It is still unknown how this may affect how the Baidu Index is used to calculate the prevalence of osteoporosis. The real search volume is not transparent and will surge abruptly with a large number of media headlines unrelated to the actual incidence rate of diseases. This is another drawback of using the Baidu Index.

Despite these drawbacks, this study demonstrates that there are numerous people who use search engines to seek information regarding "osteoporosis" and available treatments. Internet search trend data can be used as an exploratory tool to better understand patient features and priorities and is a useful source for tracking information-seeking behavior related to osteoporosis. It could assist in providing targeted online health information services, scientific proof for the management of osteoporosis, and prevention methods in China. Future studies will evaluate the dependability and stability of employing big data from the Internet as a monitoring technique.

## Conclusions

The Baidu Index can monitor Chinese online behavior and interest in health-related topics. This may enhance our understanding of disease incidence rates, population interest, resource efficiency, and patient education. The rate of online searches has continued to grow consistently, as the prevalence of osteoporosis has increased over the past few decades. Online search data analysis appears to present the geographical and demographic characteristics of osteoporosis.

## Appendices

Appendix 1

Availability of Data and Materials

The data used in this study is provided by the Baidu Index platform: (https://index.baidu.com/v2/main/index.html#/help).
